# Phase angle derived from bioelectrical impedance analysis as a marker for predicting sarcopenia

**DOI:** 10.3389/fnut.2022.1060224

**Published:** 2022-12-15

**Authors:** Haotian Wu, Ping'an Ding, Jiaxiang Wu, Peigang Yang, Yuan Tian, Qun Zhao

**Affiliations:** ^1^The Third Department of Surgery, The Fourth Hospital of Hebei Medical University, Shijiazhuang, China; ^2^Hebei Key Laboratory of Precision Diagnosis and Comprehensive Treatment of Gastric Cancer, Shijiazhuang, China

**Keywords:** bioimpedance analysis, muscle mass, muscle strength, phase angle, sarcopenia, survival

## Abstract

Sarcopenia is commonly defined as the age-related loss of muscle mass and function and may be caused by several factors, such as genetics, environmental conditions, lifestyle, drug use, and, in particular, comorbidities. People with pre-existing conditions are more likely to develop sarcopenia and subsequently have a less favorable prognosis. Recently, phase angle (PhA), which is derived from bioelectrical impedance analysis (BIA), has received a great deal of attention, and numerous studies have been carried out to examine the relationship between PhA and sarcopenia in different conditions. Based on these studies, we expect that PhA could be used as a potential marker for sarcopenia in the future.

## Introduction

Sarcopenia is a skeletal muscle disorder characterized by the accumulated loss of muscle mass and strength, and starts to develop at around 40 years of age for most sufferers (1). A recent epidemiological study found that the prevalence of sarcopenia varies between 10 and 27% across the world (2). Currently, an increasing number of studies have shown that community-dwelling people suffering from severe sarcopenia have an increased risk of adverse outcomes, such as falls, fractures (3), mobility disorders, lower quality of life, and even death (4). In addition, patients with sarcopenia have longer hospital stays and worse progression-free survival (PFS) and overall survival (OS) (5–7). In general, there are two diagnostic criteria for sarcopenia that are widely used: one is the European Working Group on Sarcopenia in Older People 2 (EWGSOP2), which uses computed tomography (CT), magnetic resonance imaging (MRI), and dual-energy x-ray absorptiometry (DXA) to diagnose sarcopenia (8), and the other is the 2019 Asian Working Group for Sarcopenia (AWGS), which uses dual-energy X-ray imaging (DXA) measurement of the appendicular skeletal muscle mass, low muscle strength (e.g., handgrip strength [HGS]), and low physical performance (e.g., walking speed) (9). Nonetheless, these complex procedures have some limitations, as they are unrepeatable and always require professional guidance. Given this, a simple, cost-effective, reliable, and reproducible biomarker is urgently needed to screen for and predict sarcopenia.

Recently, there has been growing interest in bioelectrical impedance analysis (BIA), which is a safe, non-invasive, and inexpensive bedside method for assessing body composition (10). The operating principle uses the empirical regression equation to measure resistance, which is mainly determined by the intracellular and extracellular fluid, and reactance, which is produced by the double layer of the cell membranes (11, 12). However, the universal indicators associated with BIA, which include fat-free mass (FFM) and total body water (TBW), are frequently hampered by the patients' hydration status and distribution of intracellular and extracellular water when assessing body composition in different clinical situations (12).

**Figure 1:**
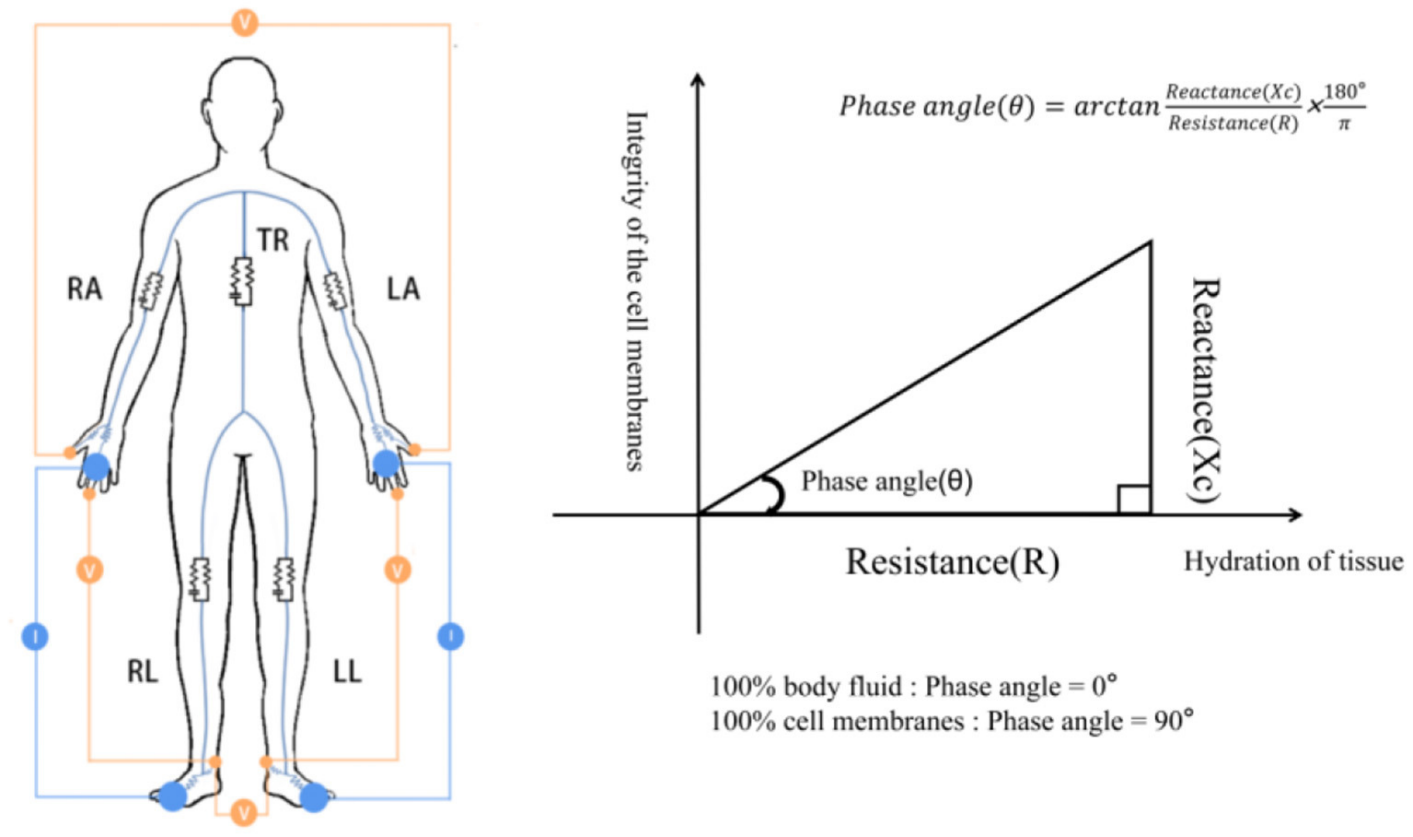
Diagram showing how PhA is measured. RA, right arm; LA, left arm; TR, trunk; RL, right leg; LL, left leg. The resistance and reactance to the voltage generated by the device is measured.

Phase angle (PhA), another raw parameter of BIA, is calculated from the original data resistive resistance (R) and capacitive reactance (Xc) by the formula arctangent (Xc/R) × 180°/π at a frequency of 50 kHz (Figure 1), and this measure is less affected by body fluid distribution (10, 12, 13). Previous studies have shown that PhA is positively correlated with cell membrane integrity and cell function. When the cell membranes are intact and the cell functions are complete PhA increases, but the situation is the opposite when the cell membranes are damaged and the selective filtration function is reduced (13–16). In healthy people, PhA has been shown to be associated with age, gender, BMI, life factors, and race (17, 18). Presently, PhA is used to predict clinical outcomes and mortality for several diseases (5, 19, 20). In addition, an increasing number of studies have considered it to be an important tool for assessing nutrition (21, 22), and it has been proposed as a possible marker for diagnosing sarcopenia, according to the 2019 EWGSOP (8). However, the validity of this parameter as a marker for predicting sarcopenia has not been evaluated.

Therefore, this review aims to summarize the role of PhA as a predictive marker for sarcopenia and explore its utility under different conditions.

## PhA prediction for sarcopenia in patients with cancer

Following extensive studies that compared patients with cancer to those without it, the former have been found to have a higher risk of developing cachexia, which can easily result in malnutrition and muscle loss, and lead to sarcopenia (6, 23). Based on the EWGSOP diagnosis criteria for sarcopenia, many studies have proved that a low PhA predicts sarcopenia risk in patients with colorectal cancer (24), gastric cancer (25), and prostate cancer (26). A cross-sectional study conducted with 124 patients in total with solid or hematologic cancer found that a low PhA is highly correlated with a high risk of sarcopenia [odds ratio (OR) = 1.74; 95% confidence interval (CI), 1.03–2.93; *P* < 0.035], after adjusting for hydration (27). A systematic review by Ji et al. involving 445 patients who were aged 65 years or older and with non-small cell lung cancer and digestive tract cancer showed that PhA was related to sarcopenia (OR = 0.309; *P* < 0.001), with a cutoff point of 4.25° (28).

In addition to the cancer types above, for which the relationship between PhA and sarcopenia has been demonstrated, there are a large number of cancers for which this relationship has not been clearly shown as the associated studies only investigated whether PhA could predict nutritional status. A systematic review, which included 16 studies of patients with breast cancer, proved that PhA can serve as a predictor of nutritional and functional status but not sarcopenia, and the predominant reason for this was that breast cancer patients were less likely to suffer from sarcopenia, resulting in an ambiguous link between PhA and sarcopenia (29). Furthermore, in patients with hepatobiliary-pancreatic (HBP) cancer (30), head and neck cancer (31), ovarian cancer (32), esophageal cancer (33), and cervical cancer (34), PhA has only been associated with malnutrition, and, to date, no studies have investigated the relationship between PhA and sarcopenia. As such, although PhA may have potential as a new prediction marker for sarcopenia in patients with cancer, further studies are needed to confirm this.

## PhA prediction for sarcopenia in patients with non-cancer diseases

Currently, a large number of studies have been carried out to evaluate whether PhA can be used a marker for predicting sarcopenia in patients with non-cancer diseases. For patients with cardiovascular diseases (CVDs), a retrospective cross-sectional analysis by Suguru Hirose et al. illustrated that PhA appears to be a useful marker for sarcopenia, and the cutoff value was 4.55° and 4.25° for males and females, respectively (35). Another study involving 310 patients with CVD found that PhA could be used to evaluate skeletal muscle damage caused by arteriosclerosis; however, only four of the patients had sarcopenia, so a relationship between PhA and sarcopenia could not be proven (36). For patients who underwent cardiovascular surgery, a significant correlation of PhA with sarcopenia was observed, demonstrating that PhA is probably a comprehensive indicator of sarcopenia (37). Overall, PhA may have a good predictive value for sarcopenia in patients with cardiac diseases.

A negative correlation between PhA and sarcopenia was observed in acute stroke patients and patients recovering from stroke; the cutoff points for sarcopenia in these instances were 5.28° for males and 4.62° for females (38), and 4.76° for males and 4.11° for females (39), respectively. A recent case series study involving 77 individuals demonstrated that for Parkinson's disease patients with sarcopenia, after adjusting for bias, only age (OR = 0.423; *P* < 0.001) was associated with PhA, but skeletal muscle mass index, grip strength, and gait speed, which were the diagnostic standards for sarcopenia, were not (40). Altogether, studies have not yet consistently shown that PhA can predict sarcopenia in patients with brain disease, and further research is needed to verify its predictive value in this context.

Meanwhile, a multicenter randomized trial involving 149 participants with chronic kidney disease (CKD) found that PhA could predict the presence of sarcopenia (*P* = 0.001) (41). A Poisson multivariate model put forward by de Amorin et al. (42), which included PhA, IL-6, and creatinine, was able to consistently predict sarcopenia in the patients with non-dialysis chronic kidney disease (ND-CKD). However, different results were obtained with kidney transplant patients. Kosoku et al. (43) found that PhA was negatively correlated with sarcopenia in kidney transplant patients, and the cutoff for predicting sarcopenia was 4.46°. A cross-sectional study involving 129 kidney transplant patients found that PhA was associated with HGS in renal transplant patients, but not sarcopenia (OR = 1.95; 95% CI: 0.71–5.39) (44). Another cross-sectional study, this time involving 346 patients who underwent maintenance hemodialysis in mainland China, found that PhA may have an optimistic predictive value for identifying sarcopenia (45). In kidney diseases, the difference is mainly concentrated in kidney transplant patients. Therefore, further research is needed to determine whether PhA can predict sarcopenia.

A study by Astrid Ruiz-Margáin, involving 413 cirrhosis patients with or without ascites, showed that PhA is lower in patients with chronic hepatitis than in patients without cirrhosis, with a cutoff value of 5.6° and 5.4° for males and females, respectively (46). Previous studies of patients with chronic obstructive pulmonary disease (COPD) (47) and peritoneal dialysis (PD) (48) have also showed that lower PhA can predict high sarcopenia risk.

Altogether, the studies above show that PhA is not a viable marker for sarcopenia in some diseases.

## PhA prediction for sarcopenia in community-dwelling people

Contemporarily, the prospect of PhA as a marker of sarcopenia risk has gained considerable popularity in community-dwelling people. Investigative research of the elderly in Japan and Poland has shown that the early risk of sarcopenia is closely related to PhA, and the optimal cutoff point for distinguishing sarcopenia from those without sarcopenia was 4.05° for males and 3.55° for females (49), and 5.42° for males and 4.76° for females (50), respectively. A study by Basile et al. (51) involving 1,567 elderly people in Italy with an average age of 76.2 (±6.7) years found that males and females with sarcopenia had a lower PhA, which was positively correlated with a reduction of muscle mass (OR = 0.623, *P* < 0.01). Two studies on elderly Mexican people also found a predictive value of PhA for sarcopenia (52, 53).

Nevertheless, a cross-sectional study performed with 94 physically active older females drew different conclusions, observing a weak correlation between low PhA and sarcopenia (OR = 1.50 (CI: 0.520–4.319; *P* < 0.01), as well as muscle mass, grip strength, and walking speed (54).

## Discussion

Based on the results above (Table 1), we find that, in terms of cancer, low PhA is associated with sarcopenia risk in patients, particularly in gastric cancer (25), colorectal cancer (24), and prostate cancer (26). However, PhA has only been proven to be associated with malnutrition rather than sarcopenia in some types of cancers (29–34). As patients with breast cancer are at lower risk of malnutrition and sarcopenia, no conclusions can be drawn on the associations between PhA and sarcopenia (29).

**Table 1:** Results of the studies with patients with different pathologies.

**Disease**	**Direction of association between PhA and sarcopenia**	**Cutoff**		**AUROC**	**Sensitivity**		**Specificity**		**Diagnostic criteria**	**Location**	**Sample**
		**Male**	**Female**		**Male**	**Female**	**Male**	**Female**			
**Cancer**											
Colorectal cancer (24)	Negative								EWGSOP	Brazil	197
Gastric cancer (25)	Negative								EWGSOP	Mexico	628
Prostate cancer (26)	Negative	4.87°		0.77					AWGS2019	Korea	119
Solid and hematologic cancer (27)	Negative	4°							SARC-F questionnaire	Brazil	124
Non-small cell lung cancer and GI cancer (28)	Negative	4.25°		0.785					AWGS2019	China	445
**Non-cancer**											
Cardiovascular diseases (35)	Negative	4.55°	4.25°	0.821/0.777	76%	61.4%	74%	86.8%	AWGS	Japan	412
After cardiovascular surgery (37)	Negative								AWGS	Japan	144
Acute stroke (38)	Negative	5.28°	4.62°	0.829					AWGS	Japan	140
Recover from stroke (39)	Negative	4.76°	4.11°	0.849/0.832	80%	73.5%	79%	82.9%	AWGS	Japan	577
Parkinson's (40)	None								EWGSOP 2019	Northeastern Brazil	77
CKD (41)	Negative								AWGS	Korea	149
ND-CKD (42)	Negative								EWGSOP 2019	Brazil	139
Kidney transplant (43)	Negative	4.46°		0.96	74%		70%		AWGS	Japan	210
Kidney transplant (44)	None								EWGSOP	Brazil	129
Maintenance hemo-dialysis (45)	Negative	4.67°	4.60°	0.82/0.83	87.93%	85.45%	69.03%	66.67%	AWGS	China	346
Cirrhosis (46)	Negative	5.6°	5.4°	0.748/0.677	94%	39%	94%	74%	SMI ≤ 50 cm^2^/m^2^ for men	American	463
									SMI ≤ 39cm^2^/m^2^ for women		
COPD (47)	Negative								EWGSOP	Italy	263
PD (48)	Negative	4.4°		0.73	81.3%		59.6%		AWGS	Korea	200
**Community-dwelling people**											
Adults of ≥50 years old (50)	Negative with pre-sarcopenia	5.42°	4.67°	0.821/0.836					EWGSOP 2019	Poland	1567
Adults of 50–64 years old (53)	Negative	4.3°		0.9306	91.95%		66.77%		EWGSOP 2019	Mexico	498
Adults of ≥65 years old (53)		4.1°		0.7930	72.76%		73.81%			Mexico	
Adults of ≥65 years old (51)	Negative								The loss of muscle mass at a rate of 1–2% per year	Italy	207
Physically active older women (54)	None								EWGSOP	Brazil	94
Women of ≥60 years old (52)	Negative								EWGSOP 2019	Mexico	250
Older adults (49)	Negative	4.05°	3.55°	0.825/0.796					AWGS	Japan	285

AUROC, area under the receiver operating characteristic; SMI, skeletal muscle index; PD, Peritoneal dialysis; COPD, chronic obstructive pulmonary disease; CKD, chronic kidney disease; ND-CKD, Non-dialysis Chronic Kidney Disease; AWGS, Asian Working Group for Sarcopenia 2019; EWGSOP, European Working Group on Sarcopenia in Older People; EWGSOP 2019, European Working Group on Sarcopenia in Older People 2019.

Moreover, we can ascertain that PhA has a strong negative relationship with sarcopenia in some non-cancer diseases (35, 37–39, 41–43, 45–48), whereas irrelevant results were found for Parkinson's (40) and kidney transplant (44) patients. Although muscle mass is reduced by prolonged paralysis in patients suffering from Parkinson's, the distribution of intracellular and extracellular water remains unchanged. Therefore, no relationship has been found between sarcopenia and PhA. As for patients who have received kidney transplants, the long-term use of immunosuppressants and hormone drugs may destroy the integrity of the cell membrane, making reactance measurement impossible, as well as sarcopenia prediction.

After comparing studies in community-dwelling people that can illustrate the negative relationship between PhA and sarcopenia with those that cannot, we speculate that the differences may be due to the sample sizes of the models (1567 vs. 94) and the different populations. Other reasons may include differences in age, sex ratios, adiposity, diagnostic methods for sarcopenia (EWGSOP vs. AWGS), measurement conditions, and equipment.

Therefore, the current research examining the utility of PhA as a marker for predicting sarcopenia has a few limitations. (1) We found that owing to the characteristics of the specific device used for measuring PhA, there may be deviations when it is measured by different devices. Additionally, there is no universal standard for the condition of the individual when measuring PhA, such as whether they are measured in the morning, whether they are measured in a fasting state, and whether they are measured while urinating, and these differences may reduce the predictive value of PhA. There are also population-specific factors that can affect PhA measurement. Therefore, when cutoff values are used to diagnose sarcopenia, researchers need to consider these factors. With this in mind, sample sizes really need to be expanded in future studies so that more accurate and reliable cutoff values can be obtained; this will allow investigation of whether sample size can change the predictive value of PhA for sarcopenia in different populations and different conditions. (2) Associations between PhA and sarcopenia were found after adjustment for hydration status in cancer patients. On this basis, as PhA can be determined by sex, age, BMI, inflammation, lifestyle factors, and the ECW/ICW ratio, we speculate that adjusting for these parameters in non-cancer situations can change the relationship between PhA and sarcopenia. (3) Both pre-disease and post-disease studies can be conducted on the same subjects to verify whether PhA can predict the occurrence of sarcopenia, and determine whether the cutoff point is the same. (4) Additionally, studies investigating whether PhA can predict pre-sarcopenia and sarcopenia are needed in the future. (5) For people with or without the disease, most of the current research still focuses on older adults over the age of 60; however, most people start to lose muscle mass and function around the age of 40 (1). Therefore, further studies are needed to determine whether sarcopenia can be predicted by PhA in middle age.

## Conclusion

In conclusion, an increasing number of studies suggest that BIA-derived PhA is an emerging and reliable predictor of sarcopenia in people with many different types of cancer; however, its association with non-cancerous conditions is still unclear. Therefore, further studies with larger sample sizes and different patient groups are required to determine the cutoff value for PhA screening for pre-sarcopenia and sarcopenia and evaluate its association with disease outcomes and prognosis.

## Author contributions

Conception and design and administrative support: QZ. Provision of study materials or patients and collection and assembly of data: PD, PY, YT, and HW. Data analysis and interpretation: PD and HW. Revise the manuscript: JW. Manuscript writing and final approval of manuscript: All authors.
